# Impact of a School Trachoma Program Emphasizing Facial Cleanliness and Environmental Improvement in Amhara, Ethiopia

**DOI:** 10.4269/ajtmh.23-0665

**Published:** 2024-05-28

**Authors:** Caleb D. Ebert, Ayenachew Kerie, Melke Kifle, Scott D. Nash, Zerihun Tadesse, Abebe Fissha, Berhanu Melak, Kassa Bulcha, Melak Haileleule, Awoke Dagnew, Ewnetu Bazie, Mitiku Adugna, Elizabeth Kelly Callahan, Mulaw Abebe, Kimberly A. Jensen, Eshetu Sata

**Affiliations:** ^1^ Francis I. Proctor Foundation, San Francisco, California;; ^2^ The Carter Center, Addis Ababa, Ethiopia;; ^3^ Amhara Regional Bureau of Education, Bahir Dar, Ethiopia;; ^4^ The Carter Center, Atlanta, Georgia

## Abstract

The SAFE (Surgery, Antibiotics, Facial cleanliness, Environmental improvement) strategy is the WHO’s endorsed approach for eliminating trachoma as a public health problem; however, not all components have been treated equally. Historically, the F and E components have not been prioritized owing to their perceived complexity. With school enrollment increasing in Ethiopia, development of a national school health program that is focused on the F and E components represents an opportunity to strengthen the SAFE strategy in the country. In 2016, the Trachoma Control Program in Amhara, Ethiopia, along with its partners, developed a School Trachoma Program (STP) that offers grade-specific lessons to improve sanitation and hygiene knowledge and practices among primary school–aged children. To assess its impact, schools were sampled before implementation and then up to 1 year after STP rollout. The aim of this report is to detail STP outcomes and the associations between outcomes and school-level variables. By 2018, adoption of an STP was strong within Amhara, with 85% of the 137 surveyed schools completing their quarterly reports and nearly 80% having at least one teacher trained in the STP. By the end of the third quarter, nearly all schools (86%) had access to a latrine, and 89% of students had a clean face. A schoolwide orientation was associated with increased STP lessons and activities (*P* = 0.01). Development of an STP, with buy-in from principals and teachers, represents a promising approach for the adoption of a new F- and E-specific curriculum and may help advance efforts to eliminate trachoma.

## INTRODUCTION

Trachoma, the leading cause of infectious blindness worldwide, is endemic in 40 countries, where an estimated 115 million people are currently living.^1^ An estimated 1.5 million people are at risk for trachomatous trichiasis – related blinding.^1^ The WHO recommends the SAFE (Surgery, Antibiotics, Facial cleanliness, Environmental improvement) strategy for the elimination of trachoma as a public health problem. Despite the strategy’s four-pronged approach, the S and A components traditionally have received more attention from researchers and public health practitioners.^2^^–^^4^ These components have elicited concrete interventions and metrics, such as the number of people operated on and the number of antibiotics distributed, which make defining progress and success easier. The F and E components have traditionally received less support, as they are more challenging to implement and monitor and are often quite costly.^5^ Interventions related to F and E are context-specific and often require collaboration with other organizations, such as those working in water and sanitation, rural development, and education, and/or other government entities to implement. Despite the difficulties associated with implementing F and E interventions, these components may be the key to ensuring sustained low levels of trachoma once elimination as a public health problem has been reached.^6^

Hygiene and facial cleanliness interventions are often tailored to preschool- and primary school–aged children.^2^^,^^7^ Children in this age group are most likely to be infected with ocular *Chlamydia trachomatis*, the causative bacterium for trachoma, potentially serving as a reservoir for their communities.^8^ Primary schools provide an excellent opportunity to promote facial cleanliness and improved hygiene practices on an ongoing basis among this at-risk population, as unclean faces attract eye-seeking flies that transmit the bacterium. Increasing facial cleanliness and improving hygiene have been shown to reduce a child’s susceptibility to trachoma; thus, grounding school programs in these interventions could decrease transmission of trachoma among students and possibly their communities.^9^ In addition, attendance at school has already impacted the behavior of students as it relates to face washing with soap; there is an opportunity to reinforce and build upon this practice as it relates to trachoma prevention.^10^ By targeting primary schools, hygiene interventions could indirectly reach younger children, as school-aged siblings may bring hygiene lessons home.

The Amhara Regional Health and Education Bureaus began adding F and E components to the region’s primary school curriculum in 2001 and had reached all districts in the region by 2008. The regional scale-up of F and E in primary schools coincided with a wider push of the full SAFE strategy to all districts in 2007. This push came as a result of survey data indicating the entire Amhara region was endemic for trachoma.^11^ After approximately 5 years of the full SAFE strategy, results from trachoma impact surveys continued to demonstrate that many districts remained highly endemic.^12^^,^^13^ Furthermore, it was determined that low levels of facial cleanliness and water availability were associated with trachoma hotspots throughout the region.^14^ With an increasing school enrollment rate in Ethiopia, from 40.2% in 2000 to 84.6% in 2015, strengthening of school-based interventions to promote hygiene and facial cleanliness presented a prime opportunity to raise awareness of trachoma and promote its elimination through public health activities.^15^ In 2015, the Trachoma Control Program in Amhara began development of a robust School Trachoma Program (STP).

The aim of this report is 3-fold: first, to detail the development of an STP focused on the F and E components of trachoma elimination; second, to describe the program’s monitoring and evaluation framework; and third, to report associations between STP outcomes and school-level variables 1 year after implementation. These results will allow for a clear assessment of the feasibility and sustainability of such a program across a large trachoma-endemic region.

## MATERIALS AND METHODS

### Developing a primary-school STP.

A Behavior Change and Communication Evaluation (BCCE) survey was conducted in all 10 zones (defined as an administrative unit made up of many districts) of Amhara in 2015 to inform the design of a regional primary school trachoma curriculum. This evaluation assessed individuals’ determinants that influenced their hygiene and sanitation practices (e.g., face washing, latrine usage, handwashing) and identified opportunities for messaging and implementation of hygiene and sanitation tools.^16^ Based on the results of the BCCE survey, the Amhara Bureaus of Health and Education, The Carter Center, and the University of California, San Francisco developed grade-specific trachoma curricula for primary school grades 1–4 that emphasized the F and E components of the SAFE strategy.^17^ These lessons were developed to fit into the existing regional primary school curricula and were aligned with existing messages on health in each grade-level syllabus. This integration with existing school curriculum was done to ensure acceptability by the educational system and increase uptake by teachers. Each grade-specific curriculum was intentionally designed to motivate students to use their gained knowledge in everyday life and encourage them to teach this knowledge to their family members, neighbors, and children who do not attend school.

The four lessons included in each grade-level curriculum were 1) what is trachoma; 2) transmission and prevention of trachoma; 3) personal hygiene and environmental sanitation (including face washing and latrine use); and 4) behavioral change activities in and out of the school compound. The STP curriculum included a teacher’s manual that provided detailed, 40-minute lesson plans for each of the four trachoma topic areas; corresponding visual aids provided examples of how to teach students about trachoma and the SAFE strategy and included resources and tools to promote trachoma elimination activities at their school. Activities included the establishment of active anti-trachoma clubs, defined as clubs that have a trachoma activity plan and are actively doing tasks that can be verified by minutes and reports. Clubs were often established and supported though collaborations with local parent-teacher associations (PTAs). The STP also encouraged local health extension workers to teach lessons within the school. All materials were finalized in English and then translated into Amharic; they were later translated into Oromiffa, Himtegna, and Agewugna to cover all major languages in the Amhara region (curricula are available at https://www.cartercenter.org/health/trachoma_education/mtResources.html).

### STP implementation.

Training for STP implementation began in late 2016 in the five zones in East Amhara and was extended to the remaining five zones in West Amhara in 2017. During the 3-day training of trainers (ToT) session, 194 trainees in East Amhara and 253 trainees in West Amhara were trained using the ToT manual. Trainees were educators selected based on an educational background in environmental science, experience in health education campaigns, and exposure to health programs and participation in school club activities. After the training, the trainees conducted cascade trainings with more than 8,300 primary school teachers and another 8,300 school directors throughout the region. All trained teachers received the ToT manual and were encouraged to conduct orientation sessions with the other teachers in their schools to expand the STP’s impact. The STP was officially launched regionwide in September 2017.

### Monitoring and evaluating the STP.

A cross-sectional baseline assessment of all 8,384 primary schools in Amhara was conducted between March and June 2016, prior to implementation of the STP. Cluster supervisors, who supported and supervised three to five schools within their catchment areas, served as data collectors after receiving a 1-day training. Zone and district education experts supervised the cluster supervisors during data collection. Data collection in each school consisted of a desk review of school documents to determine the number and composition of students and teachers in the school, the existence and activity of a school anti-trachoma club, a direct observation of facial cleanliness of students, the presence and condition of latrines, and the hand/face washing facilities in the school.

Facial cleanliness was defined as a dichotomous outcome. An unclean face was defined as having discharge and/or flies hovering around the eyes. A clean face was defined as having no discharge or flies around the eyes. A latrine was defined as functional if it had a pit that was not full, had no holes other than a squatting seat, had walls without holes or considerable damage, was fully covered by a roof to prevent direct sunlight and rain, had properly fitting doors that could be easily opened and closed, and was currently in use by the school. All school-level data were verified for accuracy by the school director.

#### Quarterly monitoring.

Stakeholders responsible for developing and deploying the STP also created a robust monitoring platform on which to assess program uptake and implementation on a quarterly basis. Primary schools were provided with monitoring registration books, in which trained teachers or directors entered data on a series of metrics regarding the frequency and number of STP lessons taught, who taught the lessons, results from facial cleanliness assessments (conducted by trained teachers), the presence of latrines, and the presence and activity of an anti-trachoma club. Logbook reports were submitted by schools quarterly and reviewed by focal person(s) at the district-level education office; carbon copy reports were shared with the relevant zonal education office and The Carter Center.

#### One-year follow-up assessment of primary schools.

**Figure 1.**
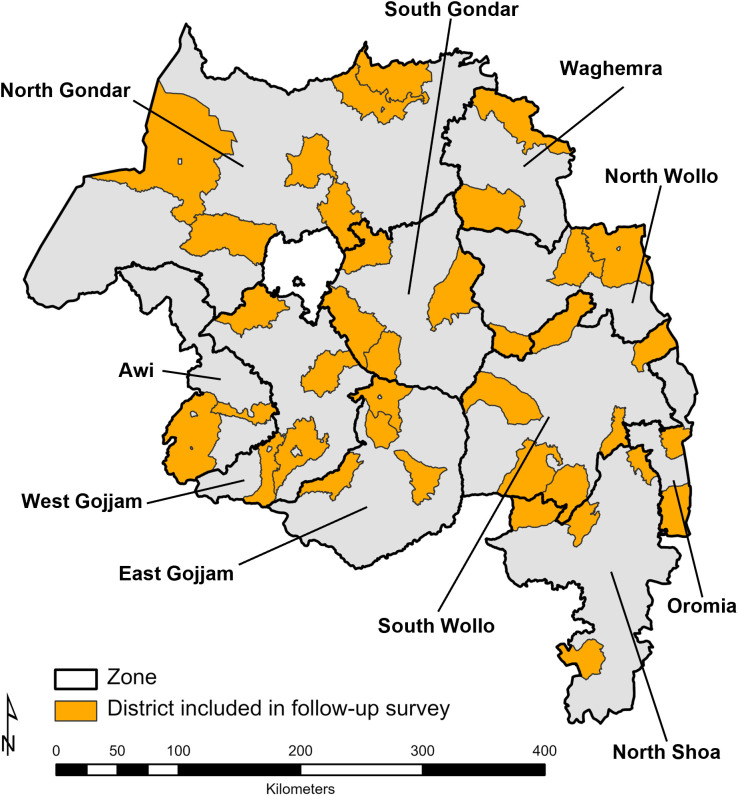
Geographic location of districts with schools that participated in the School Trachoma Program’s 1-year follow-up assessment, Amhara, Ethiopia, 2018.

After the implementation of the STP, a 1-year follow-up cross-sectional survey was conducted in June 2018 to assess the status and uptake of the program in Amhara. To establish the sample size for the survey, the percentage of regional schools reporting trachoma health programming in the recent quarter was assumed to be 77% (±10%), with a design effect of 2; thus, 137 schools were targeted. To reach this sample size, a multistage random sampling approach was used to select schools, in which districts within zones were selected randomly (Figure 1) in the first stage and schools within the selected districts were randomly selected in the second stage. The number of schools sampled in each zone was proportional to the total number of eligible schools in that zone.

Similar data collection methods that were used in the baseline assessment were used in the follow-up assessment, including a review of the STP materials present at the selected schools and an observation of the latrines and hand/face washing facilities. The existence of an anti-trachoma club was recorded, as was whether the club was active, defined as a club that had a trachoma activity plan and was actively engaging in tasks of the club that could be verified by minutes and reports. Information was captured using questionnaires completed by data collectors who had received a 1-day training prior to conducting their evaluation.

## STATISTICAL ANALYSES

Survey data were double entered in Microsoft Excel, and all data cleaning and analyses were completed in Stata (v. 15; Stata Corp, College Station, TX). Descriptive statistics (means and ranges) were calculated for each STP assessment variable. Pearson correlation coefficients were calculated using the *pwcorr* package to test for significance between the completion of an STP activity and a school-level variable. Three school-level variables of interest were considered: a school having at least one teacher trained in the STP curriculum (“trained teacher”), a school having a trained principal (“trained principal”), and a school holding at least one trachoma orientation for its teachers (“school orientation”).

## RESULTS

A total of 8,384 primary schools in all 10 zones of Amhara were initially surveyed prior to starting the STP (Table 1). Among the schools surveyed, 62.9% (zonal range: 42.4–86.2%) had a functioning latrine, and 20.3% (zonal range: 9.3–47.1%) had access to water for hand/face washing. One in every four schools, or 25.5% (zonal range: 6.5–69.8%), had a school anti-trachoma club available for its students.

**Table 1:** Baseline (2016) and 1-year (2018) school-level assessment results, Amhara, Ethiopia

Zone	Baseline (year)					1-Year Assessment				
	Schools	Functional Latrines	Separate Latrines for Males and Females	Water Available for Hand and Face Washing	Existence of Anti-Trachoma Club	Schools	Functional Latrines	Separate Latrines for Males and Females	Water Available for Hand and Face Washing	Existence of Anti-Trachoma Club
	*N*	*n* (%)	*n* (%)	*n* (%)	*n* (%)	*N*	*n* (%)	*n* (%)	*n* (%)	*n* (%)
Awi	507	354 (69.8)	185 (36.5)	239 (47.1)	354 (69.8)	6	6 (100.0)	0 (0.0)	4 (66.7)	6 (100.0)
East Gojjam	895	680 (76.0)	248 (27.7)	165 (18.4)	58 (6.5)	16	15 (93.8)	8 (50.0)	10 (62.5)	16 (100.0)
North Gondar	1,588	833 (52.5)	289 (18.2)	170 (10.7)	126 (7.9)	24	19 (79.2)	8 (33.3)	5 (20.8)	13 (54.2)
South Gondar	1,011	689 (68.2)	289 (28.6)	212 (21.0)	594 (58.8)	16	13 (81.3)	1 (6.3)	2 (12.5)	13 (81.3)
West Gojjam	921	794 (86.2)	431 (46.8)	227 (24.6)	184 (20.0)	16	15 (93.8)	9 (56.3)	8 (50.0)	15 (93.8)
North Shoa	1,029	651 (63.3)	361 (35.1)	236 (22.9)	285 (27.7)	16	16 (100.0)	7 (43.8)	10 (62.5)	15 (93.8)
North Wollo	726	433 (59.6)	265 (36.5)	172 (23.7)	164 (22.6)	16	13 (81.3)	3 (18.8)	5 (31.3)	14 (87.5)
Oromia	257	109 (42.4)	72 (28.4)	24 (9.3)	30 (11.7)	4	3 (75.0)	3 (75.0)	1 (25.0)	4 (100.0)
South Wollo	1,197	613 (51.2)	366 (30.6)	222 (18.5)	228 (19.0)	19	15 (78.9)	9 (47.4)	6 (31.6)	15 (78.9)
Waghemra	253	116 (45.8)	68 (26.9)	32 (12.6)	117 (46.2)	4	0 (0.0)	0 (0.0)	4 (100.0)	4 (100.0)
Total	8,384	5,272 (62.9)	2,575 (30.7)	1,699 (20.3)	2,140 (25.5)	137	116 (84.7)	48 (35.0)	55 (40.1)	115 (83.9)

As part of the STP, all schools were asked to complete quarterly reports regarding their implementation progress. Reporting compliance increased with each subsequent quarter, culminating in 85% of schools providing a quarterly report at the end of Quarter 3 (Table 2). Schools reported that most students had a clean face (88–90%) and had access to a latrine (85–88%) across each of the three reporting periods.

**Table 2:** Quarterly monitoring reports of STP implementation in primary schools, Amhara, Ethiopia, 2018

Description	Baseline*	Quarter I	Quarter II	Quarter III
		September–December	January–March	April–June
		2017	2018	2018
Total Schools Expected for Reporting^†^	–	7,737	8,330	8,350
Schools Reporting STP Activities, *n* (%)	–	5,959 (77)	6,872 (82)	7,066 (85)
Students Who Received Health Education in Classrooms (*n*)^‡^	–	1,591,588	2,021,793	2,215,859
Students Who Were Taught Health Education by HEWs (*n*)^‡^	–	931,289	1,223,180	1,419,813
Students Assessed for Clean Face (*n*)^§^	822,326	1,535,770	1,955,978	2,037,129
Students with a Clean Face, *n* (%)	546,321 (66)	1,374,628 (90)	1,716,841 (88)	1,805,846 (89)
Schools with Functional Latrines, *n* (%)	5,272 (63)	6,809 (88)	7,080 (85)	7,181 (86)
Schools with Handwashing Facilities, *n* (%)	1,699 (20)	3,327 (43)	3,332 (40)	3,758 (45)
Schools Reporting Active Anti-Trachoma Clubs, *n* (%)^‖^	2,140 (26)	6,963 (90)	7,663 (92)	7,682 (92)

HEW = health extension worker; STP = School Trachoma Program.

* Based on the baseline assessment of schools (*N* = 8,384) conducted by The Carter Center staff. Quarterly reports from the teachers and directors of the schools themselves.

^†^ Based on revisions to eligible list according to new schools or nonfunctioning schools.

^‡^ Numbers reported are not unique students but reflect the cumulative number of students receiving each lesson in the curriculum.

^§^ Numbers reported are not unique students but reflect the cumulative number of students being assessed for a clean face as part of the STP.

^‖^ Defined by schools reporting a meeting of the trachoma club during the quarter.

One year after implementation of the STP, a random sample of 137 schools were surveyed again. The percentage of schools with a functioning latrine and access to water for hand/face washing was 84.7% and 40.1%, respectively. The presence of a school anti-trachoma club was 3 times as high (83.9%) in the sample of resurveyed schools at 1 year compared with the full set of schools at baseline. Among the 137 schools, 109 schools, or 79.6% (zonal range: 68.8–100.0%), had trained at least one teacher in trachoma, and 58.4% (zonal range: 25.0–83.3%) reported teaching STP lessons (Supplemental Table 1). However, only 34.3% (zonal range: 0.0–66.7%) of these schools were documenting their teachers’ lesson plans in the register. Weekly facial cleanliness assessments were conducted by teachers in 32.8% (zonal range: 18.8–66.7%) of the schools, and just 8.0% (zonal range: 0.0–18.8%) had health extension workers visiting the classroom once a month to teach.

Among the schools surveyed at 1 year, correlations were evaluated between the three training metrics (having an STP-trained teacher, having an STP-trained principal, and holding the recommended STP orientation for the school’s teachers) and a school’s metrics of STP uptake. Statistically significant correlations existed only between the implementation of schoolwide STP orientation and STP uptake (Table 3). School STP orientation was positively associated with the presence of a functional latrine at the school (*r* = 0.353; *P* <0.001), the teaching of STP lessons (*r* = 0.212; *P* = 0.01), the presence of an anti-trachoma club at the school (*r* = 0.210; *P* = 0.01), having a regular environmental sanitation activity at the school (*r* = 0.202; *P* = 0.02), and the school reporting STP activities in its quarterly reports (*r* = 0.183; *P* = 0.03). Additional positive trends were observed with schools with trained principals, though the relationships were not statistically significant. No correlations were observed between successful implementation of STP and having an STP-trained teacher.

**Table 3:** Correlation between teacher and principal training, school orientation, and STP activities at 1 year, Amhara, Ethiopia, 2018

Variable	Trained Teacher		Trained Principal		School Orientation	
	Corr. Coeff.*	*P*	Corr. Coeff.*	*P*	Corr. Coeff.*	*P*
STP Lessons Taught	−0.061	0.48	0.155	0.07	0.212	0.01
STP Lessons Documented	0.109	0.35	0.091	0.44	0.043	0.71
STP Activities Reported^†^	−0.040	0.64	0.116	0.18	0.183	0.03
Facial Cleanliness Assessed	0.008	0.93	0.094	0.27	0.115	0.18
Health Education Conducted	0.083	0.33	0.129	0.13	0.088	0.31
Existence of Anti-Trachoma Club Created	−0.025	0.78	0.134	0.12	0.210	0.01
Anti-Trachoma Club Active^‡^	0.041	0.66	0.093	0.32	0.049	0.60
Functional Latrines	0.023	0.79	0.011	0.90	0.353	<0.00
Available Water for Hand and Face Washing	−0.280	0.75	−0.007	0.94	0.053	0.54
Regular Environmental Sanitation Activities Conducted	−0.140	0.12	−0.680	0.43	0.202	0.02

STP = School Trachoma Program.

* Pearson’s correlation coefficient.

^†^ Reported in quarterly reports.

^‡^ Active anti-trachoma club was defined as a club that had a trachoma activity plan and was actively doing tasks of the club that could be verified by minutes and reports.

## DISCUSSION

Over the course of the first year of the program, a high uptake of STP activities (85%) by teachers and school directors was reported, allowing students in grades 1–4 in more than 8,000 schools to be exposed to important sanitation and hygiene lessons. The STP curriculum, which was developed in collaboration with local educators, was activity based and focused on trachoma knowledge and preventative behaviors such as face washing and latrine use. This curriculum was implemented within the context of increased access to functional latrines, water for hand and face washing, and the broad establishment of anti-trachoma clubs. The STP represents an important and impactful addition to the SAFE strategy interventions implemented by the Trachoma Control Program in Amhara as it seeks to eliminate trachoma as a public health problem.

Considerable time and resources were invested as part of the STP to develop locally appropriate lessons and train educators to serve as facilitators for their schools. Investments in training teachers and providing teachers with good training materials have been important for successful school-based health programs focused on tropical diseases.^18^^,^^19^ Among the schools surveyed after 1 year of STP implementation, those that conducted schoolwide orientations on the program had the only statistically significant associations with positive STP outcomes. This included a higher likelihood of a functioning latrine, the presence of an anti-trachoma club, and a regular environmental sanitation event at the school. These relationships were considerably weaker in schools that had only a trained principal or teachers compared with schools that conducted STP orientation for all school staff. These findings suggest the importance of not only proper training for school personnel but also involvement of the entire school to achieve a successful school intervention program. As the STP continues to scale up in Amhara, the focus should be on enabling STP-trained facilitators to encourage school administrators to take a schoolwide approach to adopting STP curricula.

The success of STP adoption across schools may be credited to stakeholder engagement during the iterative curriculum development process. Time after time, stakeholder engagement across all levels has been critical for wide adoption of curricula.^20^ During curriculum development, teachers and school directors were involved to enhance understanding of their needs when teaching the new curriculum, and engagement with the Amhara Bureaus of Health and Education informed the development from a policy and technical perspective, to ensure that key information was conveyed in the lessons.^16^ As suggested by other systematic reviews, this multiprong approach of including different stakeholders during the development process will lead to effective uptake and implementation of new health education curricula.^21^ With STP, involvement of stakeholders extended beyond the curriculum development stage. The ToT manual and regular engagement with teachers through supervision supported and empowered teachers to teach the lessons. This was in response to the fact that teachers mentioned the need for accompanying materials and supportive supervision during the development process. Regular engagement with schools and teachers was also made possible through the quarterly reports collected from schools, providing a regular reminder that trachoma lessons should be implemented in the classroom and throughout the school. Health extension workers were also encouraged by the Ministry of Health to be directly involved with in-class instruction.

Data from both the quarterly reports and 1-year survey suggested that school-based water, sanitation, and hygiene (WASH)–related structural improvements were occurring in the region. By 2018, the prevalence of functional latrines was greater than 80% and the presence of hand/face washing stations was approximately 45%. Some of these observed improvements may have been possible through the contributions of local PTAs, which are active in Amhara. Higher levels of WASH infrastructure likely benefitted current students, as suggested by the consistently high prevalence of clean faces observed, and will likely continue to benefit future students as well. These long-term gains are the reason that F and E interventions should be prioritized in Amhara and are also in line with the WHO’s Sustainable Development Goal 6, ensuring availability and sustainable management of water and sanitation for all by 2030.^22^ A school environment of improved water and sanitation is important for the effectiveness of health behavior education espoused by the STP and similar programs.^23^ Without the availability of items such as latrines or water points, lessons related to latrine use and face washing while in school would be nearly impossible to apply. The STP should strive to reach 100% for these important indicators; therefore, as funding for latrine and water point construction becomes available, schools in the STP program may be ideal placement sites. Although the pairing of infrastructure and targeted education within the STP is encouraging, more evidence is needed as to whether school-based interventions truly initiate and maintain desired behavioral change.^2^^,^^3^^,^^24^ The STP should consider implementing periodic knowledge, attitude, and practice surveys and structured observations to track students and possibly their families over time, including outside of the school day and calendar. The curriculum could then be adjusted as required to achieve sustained behavioral change such as effective face washing with soap and consistent latrine usage (stopping open defecation), which will be needed to prevent trachoma transmission.

The data for this report come from administrative sources and programmatic monitoring and evaluation activities. Therefore, there was no control group of schools that did not receive the STP interventions for comparison of results. Because of this, the improvements in hygiene and sanitation metrics we observed may have been due to the STP or may have been confounded by other programs that were happening within the communities and at the national school program level. As a result of collecting observational data openly, observation bias may have affected some metrics, as school directors and teachers knew in advance when STP staff were coming to observe. In addition, assessments at baseline and 1 year were conducted by STP staff, whereas all quarterly reports were completed by trained teachers and principals. Although school staff were trained on how to conduct these assessments, including facial cleanliness, there may have been variations in definition across staff members and the potential for bias in reporting. Facial cleanliness was not assessed at the 1-year survey, which would have helped validate the quarterly reporting of this outcome. For future monitoring efforts, direct monitoring by independent observers would be more appropriate to assess facial cleanliness and other outcomes. Further, although clear operational definitions of face washing are needed, the STP should strive to monitor more objective measures of behavioral change, and research is needed to determine whether school-based trachoma education interventions have a “spillover” effect in the communities surrounding the schools.^19^^,^^25^ Other limitations to consider were that not all schools completed quarterly reports, making it hard to conduct within-school comparisons across time. In addition, the 1-year survey involved a random sample of schools; thus, direct comparisons with baseline data should be done with caution.

The need for F and E programming continues to be a focus of the global trachoma community, as does the importance of sustainability. By implementing the STP as part of the existing science curriculum and collaborating with the regional health and education bureaus, trachoma prevention messaging will be regularly integrated into school programming. Further, by establishing a schoolwide approach to trachoma prevention, engaging school principals, and conducting schoolwide orientations to engage all teachers, children will learn trachoma prevention strategies in the classroom, will be reminded of such behaviors by the school anti-trachoma club, and can practice these preventive behaviors using the school-based latrines and hand/face washing stations. The overall progress observed with the STP in Amhara echoes the power of sanitation and hygiene education in primary schools observed in previous programs in Ethiopia.^26^ For example, Gelaye et al.^26^ demonstrated that targeted education alongside provision of latrines was associated with increased knowledge, attitudes, and practices toward sanitation and reduced the prevalence of trachoma within the student population. Within the Amhara Trachoma Control Program itself, these school-based F and E interventions are an exciting new complement to the already existing, community-based SAFE activities.^12^^,^^27^

As primary school enrollment continues to increase in Ethiopia and globally, schools present a great opportunity to strengthen F and E interventions through large-scale sanitation and hygiene education.^15^ As a result of the encouraging progress of the STP, which has been implemented in more than 8,300 schools in the Amhara region, the STP began to target preschool-aged children in 2022, as children in this age range harbor considerable trachoma infection in the region.^13^ The Amhara Regional Health Bureau should continue to collaborate with the educational sector to ensure sustainability. In addition, the Ministry of Health should consider partnering with the Ministry of Education to expand this approach throughout the country, as reaching and sustaining the elimination of trachoma as a public health problem in Ethiopia must be achieved in every district and region.

## Supplemental Materials

10.4269/ajtmh.23-0665 Supplemental Materials
