# Supplementary material for: Impact of a School Trachoma Program Emphasizing Facial Cleanliness and Environmental Improvement in Amhara, Ethiopia
Source: Am J Trop Med Hyg. 2024 May 28;111(3 Suppl):114–20. doi: 10.4269/ajtmh.23-0665 (PMC11376114; doi:10.4269/ajtmh.23-0665)
Supplement: Supplemental Materials [file tpmd230665.SD1.pdf]

Supplemental Table 1. School Trachoma Program additional outcomes among 137 schools surveyed, Amhara, Ethiopia, 2018

Zone	Schools	≥ 1 STP Trained Teacher		STP Lessons Taught		STP Lessons Documented		STP Activities Reported		Facial Cleanliness Assessed Weekly		Health Education Conducted by HEW		Active Anti-trachoma Club*	
	N	n	%	n	%	n	%	n	%	N	%	n	%	n	%
**Awi**	6	6	100.0	5	83.3	4	66.7	2	33.3	4	66.7	0	0.0	5	83.3
**East Gojjam**	16	11	68.8	12	75.0	5	31.3	8	50.0	6	37.5	1	6.3	15	93.8
**North Gondar**	24	19	79.2	11	45.8	5	20.8	10	41.7	9	37.5	2	8.3	12	92.3
**South Gondar**	16	12	75.0	10	62.5	5	31.3	10	62.5	4	25.0	1	6.3	11	84.6
**West Gojjam**	16	12	75.0	10	62.5	5	31.3	12	75.0	4	25.0	2	12.5	10	6.1
**North Shoa**	16	16	100.0	9	56.3	7	43.8	8	50.0	5	31.3	3	18.8	9	60.0
**North Wollo**	16	12	75.0	11	68.8	8	50.0	8	50.0	3	18.8	1	6.3	10	71.4
**Oromia**	4	3	75.0	2	50.0	2	50.0	2	50.0	2	50.0	0	0.0	3	75.0
**South Wollo**	19	14	73.7	9	47.4	6	31.6	8	42.1	7	36.8	1	5.3	12	80.0
**Wagihmera**	4	4	100.0	1	25.0	0	0.0	0	0.0	1	25.0	0	0.0	3	75.0

**Total**	137	109	79.6	80	58.4	47	34.3	68	49.6	45	32.8	11	8.0	90	65.7

* Active anti-trachoma club defined as a club that was active, defined as a club that has trachoma activity plan and is actively doing tasks of the club that can be verified by minutes and reports.
